# Impact of Chloride Impurities on the Corrosion Behavior of Stainless Steel in Molten Alkali Carbonate Salts for Concentrated Solar Power Systems

**DOI:** 10.3390/ma19071312

**Published:** 2026-03-26

**Authors:** Jing Luo, Ning Li, Naeem ul Haq Tariq, Tianying Xiong, Xinyu Cui

**Affiliations:** 1 Ocean Mechanical and Electrical Institute, Xiamen Ocean Vocational College, 4566 Hongzhong Road, Xiamen 361100, China; 2 Institute of Metal Research, Chinese Academy of Sciences, 72 Wenhua Road, Shenyang 110016, China; 3 Department of Metallurgy & Materials Engineering, Pakistan Institute of Engineering & Applied Sciences, Nilore, Islamabad 45650, Pakistan

**Keywords:** concentrated solar power, molten carbonate salt, chloride impurity, corrosion

## Abstract

This study clarifies the catalytic role of chloride ions on the corrosion performance of SS316L alloy immersed in molten LiNaK carbonate salt at 700 °C. Accordingly, isothermal static immersion corrosion tests were systematically conducted under different experimental conditions. Our results revealed that the presence of Cl significantly accelerates the corrosion process: the rate constant of the corroded samples increased from 11.3 × 10^−2^ mg/cm^2^ to 13.8 × 10^−2^ mg/cm^2^ with the addition of Cl. Continuous migration of Cl_2_ and volatile metal chlorides leads to the formation of obvious pores, transverse cracks along grain boundaries, surface wrinkles, and partial spalling of the oxide scale, thereby severely aggravating substrate degradation. Notably, no chlorine-containing compounds or chlorine-rich regions were detected in the corroded samples, confirming that chlorine is not consumed in the corrosion process, rather it acts as an autocatalyst through the cyclic process of “oxidation–diffusion–reaction–regeneration”.

## 1. Introduction

Benefiting from its inexhaustibility and high availability, solar energy is gaining escalating global focus for development and utilization [1]. Concentrated solar power (CSP) technology, an environmentally benign solar power generation approach, employs heat transfer fluids (HTFs) to achieve light-heat-electricity conversion and integrated energy storage [2]. In order to further enhance the productivity of power generation and minimize its cost, it is necessary to use HTFs with a higher operating temperature (such as carbonates or chlorides) to replace commonly used nitrates-based HTFs in current commercial CSP systems [3,4,5,6]. The ternary eutectic LiNaK carbonate salt is considered as one of the most competitive high-temperature HTFs. It has a wide operating temperature window (397–1000 °C) and has excellent thermophysical properties such as high heat capacity and high thermal conductivity [7,8,9,10].

Although molten carbonate salts display comparatively low corrosivity, the long-term corrosion resistance of structural components at elevated temperatures is a critical factor for effective CSP utilization [11]. For the past four decades, researchers have paid great attention to address the corrosion problems of molten carbonate salts used in the CSP plants [12,13,14]. After decades of development, corrosion performance of various structural materials such as steel, low alloy steel, stainless steel, and high-temperature alloys in molten carbonate salts has been investigated by various researchers [15,16,17,18,19,20]. Morales et al. [17] studied the corrosion behavior of two duplex stainless steels (DS2205 and DS2507) in Li_2_CO_3_-K_2_CO_3_-Na_2_CO_3_ molten salt in an air atmosphere at 500 °C. They found that the oxide thickness and corrosion time for DS2205 and DS2507 follows a parabolic and cubic law, respectively. Fernandez et al. [21] found that the corrosion resistance of austenitic alloys, exposed to molten ternary eutectic carbonate salt at 650 °C, is better than that of other types of stainless steels. Lambrecht et al. [18] reported corrosion performance of H230 nickel-based alloy exposed to molten Li_2_CO_3_-Na_2_CO_3_-K_2_CO_3_ in an air atmosphere at 700 °C, 750 °C, and 800 °C for 1000 h, and reported that temperature is the key regulating factor for the corrosion mechanism. A growing body of research has indicated that, in addition to temperature, parameters such as ambient atmosphere and the microstructural characteristics of alloys are also critical to the corrosion susceptibility of alloys in molten carbonate salt environments [22,23,24,25,26,27,28].

SS316L has excellent comprehensive mechanical properties and is widely used in various industries [29,30]. Researchers have extensively evaluated its suitability to be used in molten salt environments. Li et al. [23] studied the corrosion response of SS304 and SS316L in solar salt at different temperatures through electrochemical methods. They found that the corrosion of SS316L is controlled by ion transport in the oxide film, and its corrosion resistance is better than that of SS304. Liu et al. [31] explored the corrosion process of SS316 in molten NaCl-KCl-MgCl_2_ at 700 °C and reported that it is characterized by preferential corrosion at grain boundaries, and the surface oxide layer gradually evolves from a single-layer structure to a three-layer structure. Sarvghad et al. [24,25] studied stainless steels with different processing levels in LiNaK carbonate molten salt at 450 °C, and their results showed that SS316 has better corrosion resistance in the molten ternary LiNaK carbonate salt at 450 °C.

The annual demand for molten salts as HTFs in CSP systems amounts to thousands of tons, which constitutes a substantial cost driver in the overall economics of CSP installations. For this reason, CSP plants typically adopt low-cost, commercial-grade molten salts to minimize HTF-related expenses, thereby curbing power generation costs and bolstering market competitiveness [32]. Previous studies, including our team’s preliminary research, have mainly explored the corrosion response of alloys in high-purity molten carbonate salts as the corrosion medium, thereby neglecting the effects of impurities present in the low-purity molten salts used in actual CSP plants [33]. Further, some studies showed that chloride impurities in molten salts can enhance the corrosion damage of the molten salts [34,35]. Bradshaw et al. [35] studied the effect of different chloride concentrations in binary nitrate salts on the corrosion response of A516 steel by adding 0 wt.%, 0.5 wt.%, and 1.0 wt.% chloride to the Solar Salt. They revealed that the corrosion rate of the samples increased significantly with the concentration of dissolved chloride in molten nitrates, and no pitting corrosion or intergranular corrosion phenomena were observed on the corroded samples. Grabke et al. [36] employed gravimetric measurements to explore the effect of chloride on high-temperature oxidation of alloy steels at 500~700 °C, and found that chloride is the key catalyst for high-temperature active oxidation of alloy steels. However, there are few relevant reports about the corrosion mechanism of structural materials in low-purity molten carbonate salts containing chloride impurities. The corrosion behavior and the underlying corrosion mechanism of SS316L in chloride-containing molten carbonate salts remain unclear. Therefore, it is of practical significance to study the effects of chloride impurities in molten carbonate salts affecting the corrosion performance of structural materials.

To replicate practical conditions for CSP systems, where chloride impurities in commercial carbonate salts can reach ~1 wt.% [35], a eutectic LiNaK carbonate salt doped with 1 wt.% NaCl was used as the corrosion medium in this study. The corrosion behavior of SS316L was systematically examined via static corrosion tests at 700 °C, with a detailed discussion of the underlying mechanisms.

## 2. Materials and Methods

### 2.1. Materials Preparation

Commercially available AISI 316L stainless steel (TaiYuan Iron & Steel (Group) Co., Ltd., Taiyuan, China), with the chemical composition (wt.%) of Fe: Bal., Ni: 10.04, Cr: 16.71, Mo: 2.15, S: ≤0.03, P: ≤0.045, Mn: ≤2.00, Si: ≤1.0, C: ≤0.03, was used as the substrate in this study. To perform static corrosion experiments, samples measuring 25 × 25 × 5 mm^3^ were prepared by electric discharge machining. Afterward, all six faces were ground with SiC papers up to 600 grit, subjected to ultrasonic cleaning in acetone, and finally degreased with ethanol prior to subsequent experiments. A mixed salt consisting of 1 wt.% NaCl and 99 wt.% eutectic LiNaK carbonate salt (with the eutectic salt composed of 32.1 wt.% Li_2_CO_3_, 33.4 wt.% Na_2_CO_3_ and 34.5 wt.% K_2_CO_3_) was used as the corrosion medium in this research, hereinafter referred to as “LiNaK-Cl” carbonate salt in the manuscript. NaCl, Li_2_CO_3_, Na_2_CO_3_ and K_2_CO_3_ were weighed out in the aforementioned mass fractions. The weighed salts were then mechanically blended in a jar mill for 1 h, with each batch prepared to a total mass of 600 g. Finally, the homogenized mixture of salt was retrieved and vacuum-sealed for subsequent corrosion experiments. The procedure for preparing mixed salt can also be found elsewhere [33], while the characteristics of salts are summarized in Table 1.

### 2.2. Corrosion Experiment

**Figure 1:**
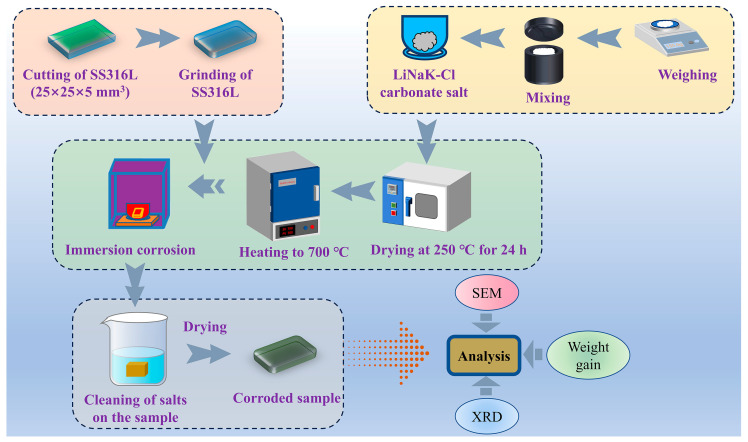
Schematic illustration of the complete experimental procedure for static corrosion testing.

Isothermal static corrosion experiments were carried out at 700 °C using a muffle furnace equipped with precise temperature control in a laboratory air atmosphere. Prior to testing, the LiNaK-Cl carbonate salt was placed in an alumina crucible and dried at 250 °C for 24 h inside the muffle furnace to eliminate moisture. The furnace was subsequently heated from room temperature to the target temperature of 700 °C at a controlled rate of 5 °C/min. Once thermal equilibrium was achieved, three samples (previously measured for surface area and initial mass) were immersed in the molten salt with strict maintenance of specimen-to-specimen separation. After exposure periods of 24, 72, 120, 168, 216, 312, 360, 408, 456, and 500 h (in order to compare with our previous corrosion experiments under chloride-free conditions [33]), the corroded samples were retrieved, allowed to cool in air to room temperature, and thoroughly cleaned with warm deionized water to remove residual salt deposits. A schematic overview of the entire corrosion testing protocol is presented in Figure 1.

Corrosion kinetics were quantified via the mass gain method. Sample masses were recorded before and after each test using an analytical balance with 10^−5^ g precision. The mass change per unit area (∆W) was calculated according to Equation (1), with the average value determined from three weight values to reduce experimental errors.

ΔW = (M_t_ − M_0_)/A_s_ (1)

where M_0_ and M_t_ represent the initial and time-dependent masses, respectively, and A_s_ denotes the initial exposed surface area.

### 2.3. Characterization Methods

Surface morphology of the corroded samples was examined using field-emission scanning electron microscopy (FESEM, FEI Apreo, Brno, Czech Republic). Elemental composition and spatial distribution were analyzed by energy-dispersive X-ray spectroscopy (EDS). Phase identification of the corrosion products was performed via X-ray diffraction (XRD, Bruker D8 Advance, Karlsruhe, Germany) employing Cu Kα radiation. To investigate phase composition within the inner corrosion layer, the outer corrosion products were carefully removed by gentle grinding with 2000-grit SiC paper (NKC, Seoul, Republic of Korea) prior to XRD analysis.

## 3. Results

### 3.1. Gravimetric Study

**Figure 2:**
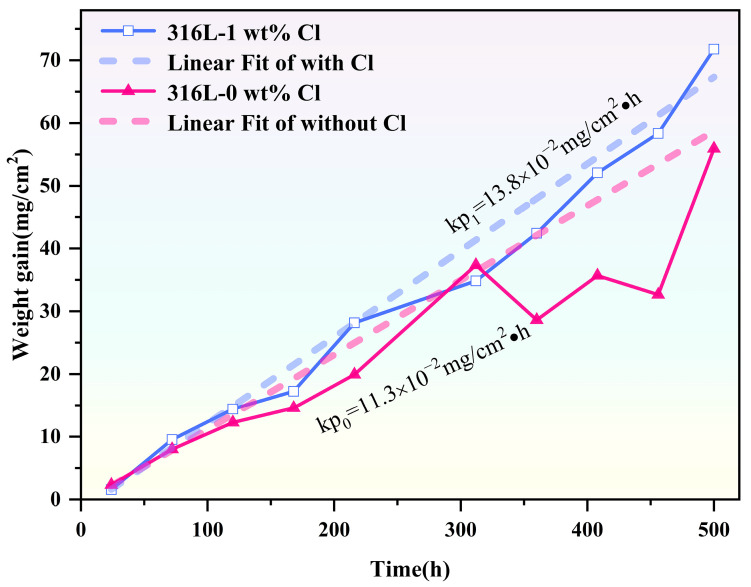
Mass gain curves depicting the corrosion kinetics of SS316L in molten LiNaK-Cl carbonate salt and chloride-free LiNaK carbonate salt at 700 °C.

Figure 2 shows the corrosion kinetic curves for SS316L samples after corrosion in molten LiNaK-Cl carbonate salt and LiNaK carbonate salt at 700 °C. As shown in the figure, both kinetic curves almost follow a linear law under the two experimental conditions. Moreover, the corrosion kinetic curve for SS316L immersed in molten LiNaK-Cl carbonate salt continuously rises throughout the corrosion process, and is notably steeper than that obtained in molten LiNaK carbonate salt. Quantitatively, the corrosion rate constant of the samples in molten LiNaK-Cl carbonate salt reaches 13.8 × 10^−2^ mg/cm^2^, while that in molten LiNaK carbonate salt is just 11.3 × 10^−2^ mg/cm^2^. The results indicate that SS316L not only underwent continuous corrosion attack in molten LiNaK-Cl carbonate salt, but also experienced a faster corrosion rate compared to that in molten LiNaK carbonate salt.

### 3.2. Sample Characterization

**Figure 3:**
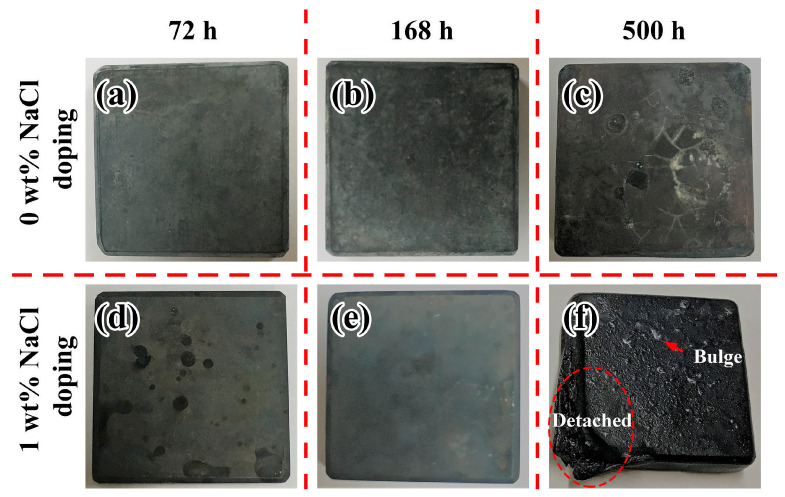
Optical images of SS316L samples after corrosion in molten LiNaK-Cl and LiNaK carbonate salt at 700 °C: (**a**) 72 h, 0 wt.% NaCl, (**b**) 168 h, 0 wt.% NaCl, (**c**) 500 h, 0 wt.% NaCl, (**d**) 72 h, 1 wt.% NaCl, (**e**) 168 h, 1 wt.% NaCl, (**f**) 500 h, 1 wt.% NaCl.

Figure 3 presents optical images of corroded SS316L samples in molten LiNaK-Cl carbonate salt and LiNaK carbonate salt at 700 °C. It can be seen that the surface oxide layers of the samples peel off under both conditions after a relatively long period of corrosion (Figure 3c,f). In addition, there are bulges on the surfaces of the samples corroded in molten LiNaK-Cl carbonate salt (Figure 3f). This might be associated with the gas generated by the influence of chloride impurity in the course of corrosion, making the corrosion layer less dense and more prone to peeling off.

**Figure 4:**
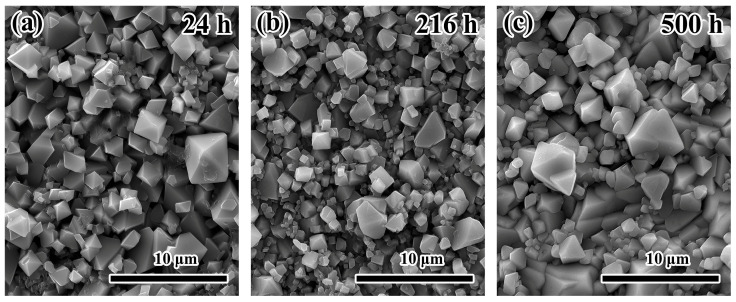
Representative surface morphologies of SS316L samples after exposure to molten LiNaK-Cl carbonate salt at 700 °C: (**a**) 24 h, (**b**) 216 h, (**c**) 500 h.

The morphologies of the corroded samples for different durations were further examined in detail using SEM. Figure 4 shows that the surface corrosion layers on all samples display a spinel structure across the full duration of the corrosion experiment.

**Figure 5:**
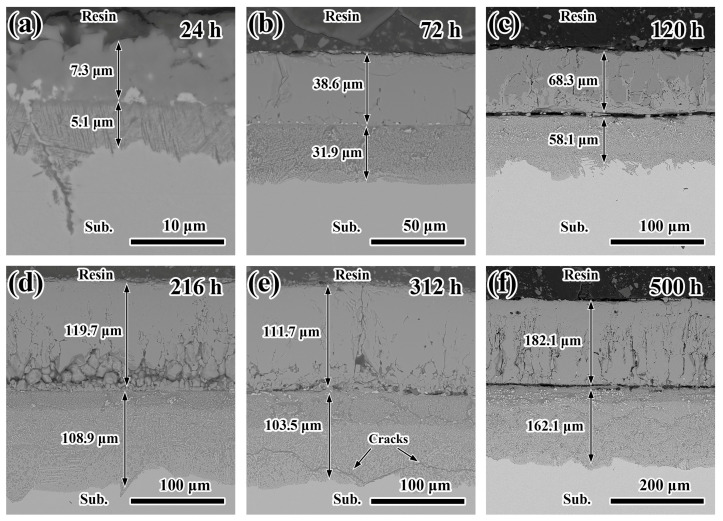
Cross-sectional SEM micrographs of SS316L samples immersed in molten LiNaK-Cl carbonate salt at 700 °C: (**a**) 24 h, (**b**) 72 h, (**c**) 120 h, (**d**) 216 h, (**e**) 312 h, (**f**) 500 h.

Figure 5 displays SEM images of cross sections of SS316L samples after immersion in molten LiNaK-Cl carbonate salt at 700 °C for different time periods. The corrosion scale exhibits a characteristic bilayer structure whose thickness grows continuously with prolonged exposure.

Furthermore, cracks appear at the interface between the inner and outer corrosion layers of the sample corroded for 120 h, as shown in Figure 5c. Transverse cracks extending along the grain boundaries are also observed in the inner corrosion layer of the sample corroded for about 312 h, as shown in Figure 5e. Interestingly, no such transverse cracks can be seen along the grain boundaries during the whole corrosion process for the samples immersed in molten LiNaK carbonate salt at 700 °C. The preferential attack along the grain boundaries, giving rise to intergranular corrosion in the SS316L samples, is a typical characteristic of localized corrosion, which might be associated with the chloride impurity. Consequently, the corrosion layer becomes less dense and easily peels off. This is quite consistent with the above experimental results, Figure 3f. On the other hand, transverse cracks within the inner corrosion layer may provide additional pathways for corrosive species to reach the underlying metals, leading to concentrated and localized damage, thus accelerating localized corrosion.

**Figure 6:**
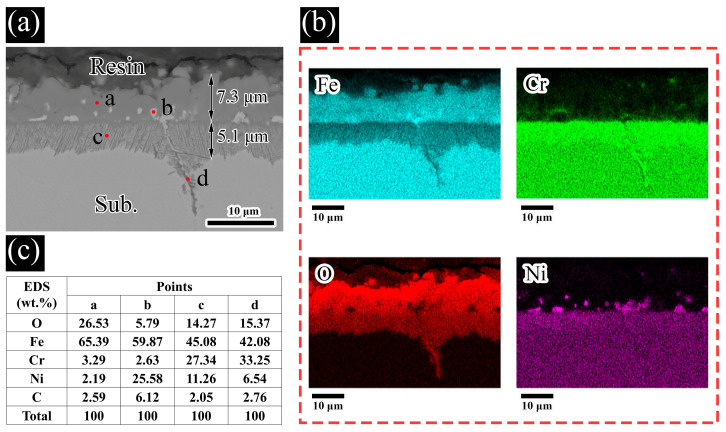
Detailed analysis of SS316L after 24 h exposure to molten LiNaK-Cl carbonate salt at 700 °C: (**a**) cross-sectional SEM image, (**b**) corresponding EDS elemental maps, (**c**) summary of EDS point analyses from panel (**a**).

**Figure 7:**
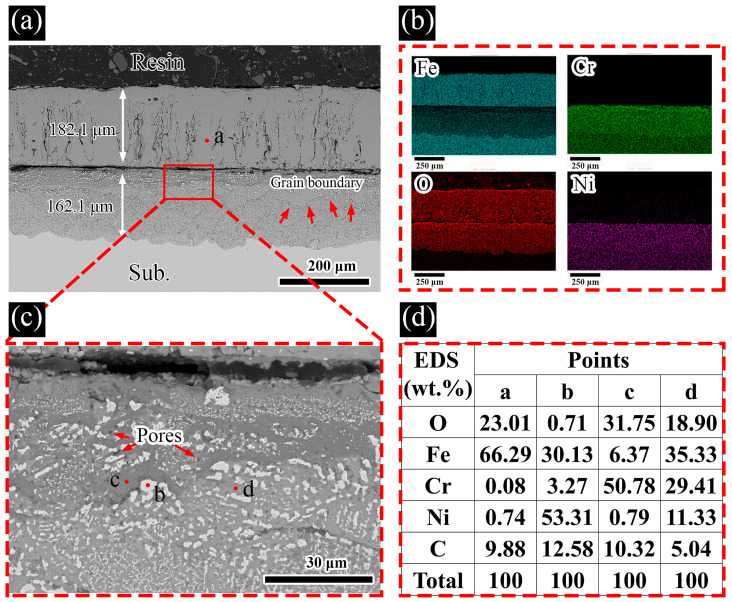
Detailed analysis of SS316L after 500 h exposure to molten LiNaK-Cl carbonate salt at 700 °C: (**a**) cross-sectional SEM image, (**b**) corresponding EDS elemental maps, (**c**) magnified view of the marked rectangular region in panel (**a**), (**d**) summary of EDS point analyses.

Figure 6 and Figure 7 display the microstructural features, elemental mappings, and compositional profiles obtained from cross-sections of SS316L samples corroded in molten LiNaK-Cl carbonate salt at 700 °C for 24 h and 500 h, respectively. Following 24 h immersion (Figure 6a), a compact bilayer corrosion product forms on the alloy surface, with inner and outer layer thicknesses ranging between 5 and 8 µm. EDS point and mapping analyses confirm that the outer layer is predominantly composed of Fe-based oxides (marked with ‘a’ in Figure 6a) along with patches of unoxidized substrate (‘b’), whereas the inner layer exhibits a complex intergrown structure of various oxide compounds.

After prolonged corrosion exposure for up to 500 h, a two-layered corrosion scale is retained on the surface of SS316L samples (as shown in Figure 7). EDS point measurements and elemental mapping analyses indicate that the outer layer is dominated by iron-based oxides, whereas the inner corrosion layer is a heterogeneous mixture of various oxide species.

The black phase (point ‘c’ in Figure 7c) is confirmed by EDS analysis to be rich in Cr and O, which is speculated to be a Cr-containing oxide. The gray phase (point ‘d’ in Figure 7c) is found to be rich in Cr and Ni with relatively high O content. The white phase (point ‘b’ in Figure 7c) is dominated by Ni and Fe elements, and is presumed to be an unoxidized substrate. In the inner area of the inner corrosion layer, the intergranular corrosion phenomenon is particularly obvious, wherein white phase and gray phase are mainly distributed around the grain boundaries. In contrast to the short-term corrosion results at 24 h, the inner and outer corrosion layers exhibit a marked reduction in density following long-term exposure for 500 h. This is clearly manifested by their distinctly loose, porous microstructures, accompanied by the formation of a clear gap at the inner/outer layer interface, leading to a dramatic deterioration in their bonding integrity. In addition, obvious micropores/defects and intergranular corrosion can be readily identified in the inner corrosion layer. It is worth noting that no obvious Cl-rich regions can be found throughout the corrosion layers of the corroded samples, and the microstructures and elemental distributions of the corrosion layers are basically similar to those exposed to molten LiNaK carbonate salt.

**Figure 8:**
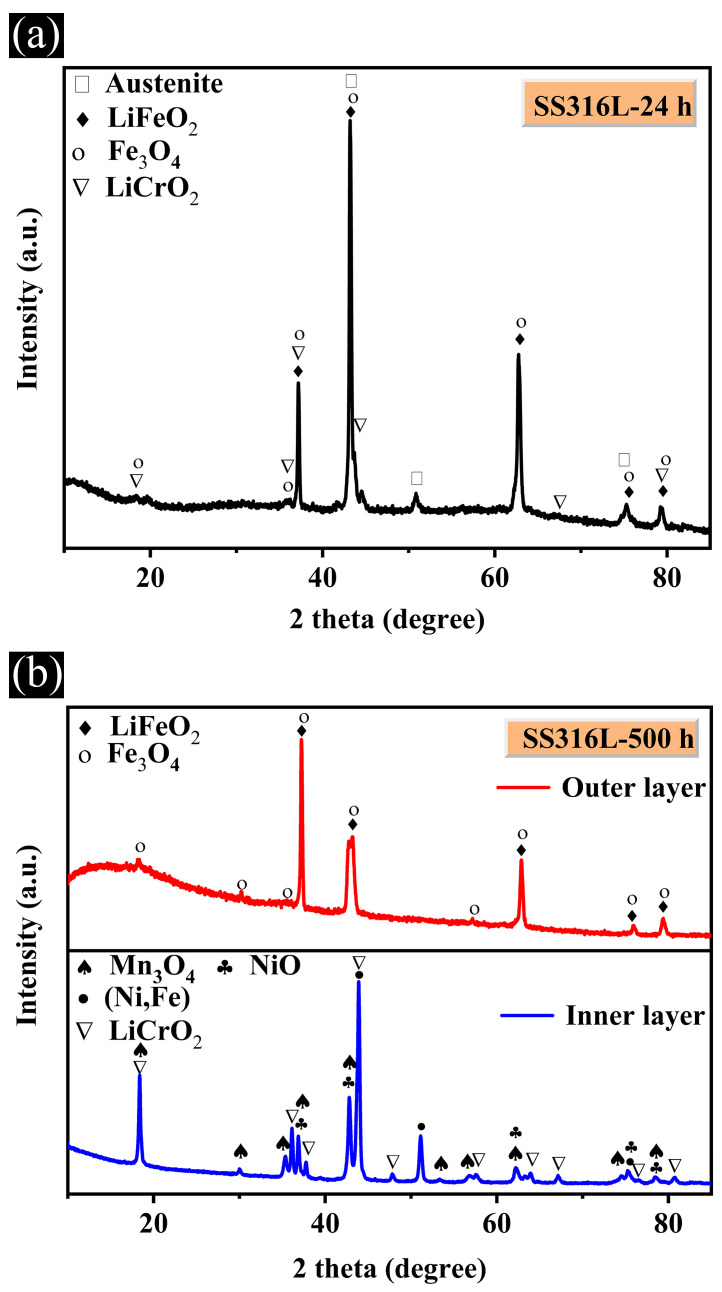
X-ray diffraction patterns of SS316L samples exposed to molten LiNaK-Cl carbonate salt at 700 °C for (**a**) 24 h and (**b**) 500 h.

The XRD results of the SS316L samples, exposed to molten LiNaK-Cl carbonate salt at 700 °C for 24 h and 500 h, are shown in Figure 8. After corrosion for 24 h, the corrosion products of SS316L samples exposed to molten LiNaK-Cl carbonate salt are mainly composed of LiFeO_2_ and a small amount of LiCrO_2_ and Fe_3_O_4_ phases (Figure 8a). In contrast, corrosion products like LiFeO_2_ and Fe_3_O_4_ are found on the sample surface exposed to molten LiNaK-Cl carbonate salt for 500 h (Figure 8b). Further, phase analysis of the inner layer of samples corroded for 500 h was performed by removing the outer corrosion layer. It can be seen that the inner corrosion layer is mainly composed of LiCrO_2_, (Ni, Fe), NiO and Mn_3_O_4_ (Figure 8b). In summary, no chlorine-containing corrosion products can be detected in either the outer or inner corrosion layer, which is consistent with the aforementioned EDS results.

## 4. Discussion

The above experimental results show that the corrosion kinetics of SS316L are substantially enhanced in molten LiNaK-Cl carbonate salt compared to the chloride-free counterpart, yet the morphological and compositional features of the corrosion products remain highly similar. To clarify this phenomenon and reveal the underlying mechanism, this section will conduct an in-depth discussion on the corrosion behavior.

It is worth noting that the high-temperature corrosion process in molten salts is essentially electrochemical in nature, involving anodic metal dissolution and cathodic reduction reactions, which provides a fundamental basis for the subsequent analysis of the corrosion mechanism.

**Figure 9:**
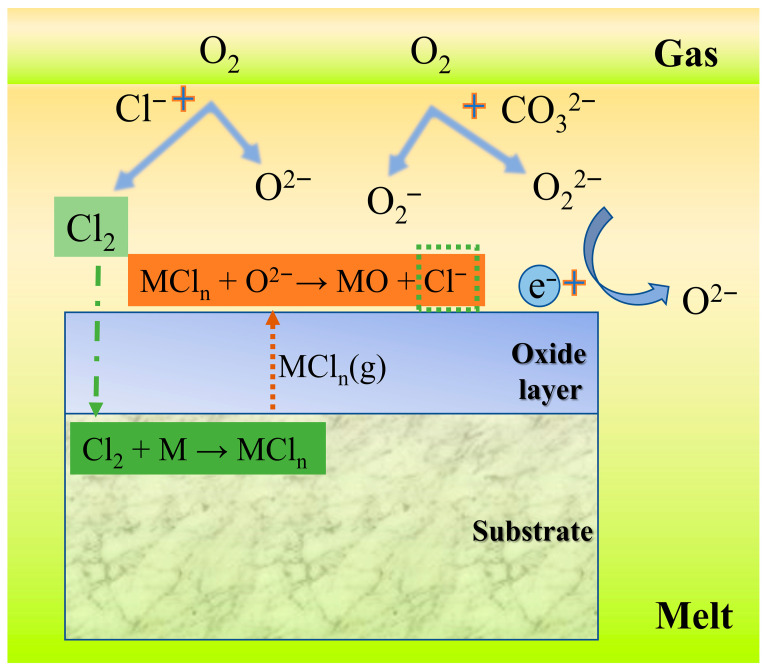
Corrosion mechanisms for alloys in molten LiNaK-Cl carbonate salt.

Among numerous studies on high-temperature corrosion of chloride salts, the “active oxidation” mechanism is widely recognized by researchers [34,36]. Combining the corrosion mechanisms of chloride salts, molten carbonate corrosion characteristics, and present experimental results, we propose the following corrosion mechanisms for alloys exposed to molten LiNaK-Cl carbonate salt (Figure 9).

In molten carbonate salt containing chlorides, the Cl^−^ in the melt will react with O_2_ at the melt/gas interface to form Cl_2_, as demonstrated by reaction (2) [38]:

2Cl^−^ + 1/2O_2_ → Cl_2_ + O^2−^ (2)

The Cl_2_ released by the reaction will dissolve in the melt and diffuse in the direction of low oxygen activity. Due to the strong penetrability of Cl_2_, it will pass through the loose oxide layer to reach the oxide layer/substrate interface, and react with metals (such as Fe, Cr) in the substrate to form metal chlorides, as indicated by reactions (3)–(5) [32,39]:

Fe + Cl_2_ → FeCl_2_(s) (3)

2Fe + 3Cl_2_ → 2FeCl_3_(s) (4)

2Cr + 3Cl_2_ → 2CrCl_3_(s) (5)

In a molten salt at 700 °C, the vapor pressure of metal chlorides at the interface is relatively high and thus the volatility is strong, and the following reaction thus occurs [32]:

MCl_n_(s) → MCl_n_(g) (6)

The volatilized metal chlorides will diffuse rapidly to the oxide layer/melt interface with high oxygen activity. Owing to the abundance of peroxide ions (O_2_^2−^), superoxide ions (O_2_^−^), and oxygen ions (O^2−^) in the molten carbonate salt [33], these metal chlorides will react with the oxygen-containing ions in the melt to form corresponding metal oxides, as illustrated in reactions (7)–(9) [33]. Additionally, the Cl^−^ released during these reactions is recycled back to the molten salt, where it is re-oxidized to Cl_2_ and repeatedly participates in accelerating the corrosion process.

2FeCl_3_ + 3O^2−^ → Fe_2_O_3_ + 6Cl^−^ (7)

FeCl_2_ + 2FeCl_3_ + 4O^2−^ → Fe_3_O_4_ + 8Cl^−^ (8)

2CrCl_3_ + 3O^2−^ → Cr_2_O_3_ + 6Cl^−^ (9)

Further, the resulting metal oxides interact with Li^+^ and O^2−^ from the molten carbonate salt, producing Li-based compounds through the following reactions [33]:

Fe_2_O_3_ + 2Li^+^ + O^2−^ → 2LiFeO_2_ (10)

Cr_2_O_3_ + 2Li+ + O^2−^ → 2LiCrO_2_ (11)

Therefore, SS316L undergoes corrosion not only from the molten carbonate salt but also experiences accelerated degradation due to the presence of chlorides in molten LiNaK-Cl carbonate salt.

The Cl^−^ in the salts will be continuously oxidized to form Cl_2_, which diffuses to the oxide layer/substrate interface, inducing the formation of metal chlorides, thereby providing more metal cations for the formation of metal oxides. Consequently, the corrosion process is accelerated. This acceleration is quantitatively verified by our experimental results: the corrosion rate constant of SS316L in molten LiNaK-Cl carbonate salt is 13.8 × 10^−2^ mg/cm^2^, which is about 22% higher than that in chloride-free molten LiNaK carbonate salt (11.3 × 10^−2^ mg/cm^2^). During this process, Cl_2_ and volatile metal chlorides continue to diffuse and migrate through the oxide layers, causing the inner and outer corrosion layers to be poorly consolidated and porous. This in turn promotes the further migration of Cl_2_ and metal chlorides within the oxide layers, thereby accelerating the formation of corrosion products. In addition, the migration of these chlorides may also impair the adhesion of the oxide layers to the substrate, resulting in transverse cracks within the corrosion layer, causing wrinkles/cracks in the surface oxide layer, and even partial peeling off of the oxide layers. In the absence of a protective oxide layer, molten salts come into direct contact with the substrate, significantly exacerbating corrosion of the base material.

In this process, the chlorine participating in the corrosion reaction is not consumed and does not form stable, detectable enrichments in the corroded samples, but only plays an autocatalytic role, accelerating the corrosion process and affecting the structures of the corrosion layers. As a consequence, no obvious Cl-rich areas or chlorine-containing compounds can be detected in the samples exposed to molten LiNaK-Cl carbonate salt.

## 5. Conclusions

To mimic the realistic conditions of commercial-grade carbonate salts used in CSP plants, 1 wt.% NaCl was added to the eutectic LiNaK carbonate salt (referred to as “LiNaK-Cl” carbonate salt). Prolonged static immersion experiments were conducted on SS316L samples in this chloride-containing molten salt at 700 °C to evaluate the effect of chloride impurities on corrosion kinetics and mechanisms. The key findings of the present study, along with their implications for material selection of CSP molten salt systems, are summarized as follows:

1. Exposure of SS316L to molten LiNaK-Cl carbonate salt at 700 °C resulted in substantially faster corrosion rates than those observed in pure LiNaK carbonate salt at the same temperature. Chloride ions were found to strongly promote corrosion progression.
2. A distinct two-layered corrosion product scale formed on the SS316L samples immersed in molten LiNaK-Cl carbonate salt. The overall thickness of this bilayer scale grew continuously with increasing corrosion time, reflecting the sustained and accumulating nature of the corrosive attack.
3. Transverse cracks along the grain boundaries and micropores were observed in the inner corrosion layer of the SS316L samples immersed in molten LiNaK-Cl carbonate salt at 700 °C. Furthermore, no Cl-rich regions were observed throughout the corroded samples, which is consistent with the proposed mechanism involving transient participation of Cl^−^ during the corrosion process.
4. Continuous outward migration of Cl_2_ and metal chlorides weakens the adhesion of the corrosion layers, resulting in prominent pores, transverse cracks, wrinkling, and partial peeling of the oxide scales, which in turn accelerates corrosion of the underlying substrate.
5. During the corrosion process, the Cl^−^ participating in the reaction was not consumed, but exerted an autocatalytic effect through the cyclic process of “oxidation-diffusion-reaction-regeneration”. This is also the core reason why no Cl-containing compounds or Cl-rich areas were detected in the corroded samples.
6. Given the severe, sustained corrosive attack endured by SS316L in molten LiNaK-Cl carbonate salt, bare SS316L without protective coating is not viable for application as a structural component in CSP molten carbonate salt systems.

In summary, this study systematically clarifies the corrosion acceleration mechanism of chloride impurities and the corresponding degradation behavior of SS316L in molten LiNaK-Cl carbonate salt at 700 °C, which are critical for guiding the reliable operation of CSP systems. The identification of chloride’s autocatalytic cycle provides a fundamental theoretical basis for understanding impurity-driven corrosion in high-temperature molten salt environments. Practically, these findings highlight the limitations of bare SS316L in chloride-containing carbonate systems. Future research could further focus on screening and optimizing coating materials that possess excellent resistance to chloride corrosion and stable performance under the high-temperature conditions of CSP systems.

## Figures and Tables

**Table 1:** Characteristics of Li_2_CO_3_, Na_2_CO_3_, K_2_CO_3_ and NaCl. (The thermophysical properties are taken from reference [37]).

Salt	Molecular Mass g/mol	Heat of Fusion J/g	Melting Point °C	Purity %	Impurity Level (Max) ppm			
					Ca	Cl	N(NO_3_)	SO_4_^2−^
Li_2_CO_3_	73.9	509	732	98.0	300	50	30(NO_3_)	500
Na_2_CO_3_	106.0	165	858	99.8	100	20	10(N)	50
K_2_CO_3_	138.2	202	900	99.0	20	30	10(N)	30
NaCl	58.4	482	801	99.5	20	-	5(N)	10

## Data Availability

The raw/processed data required to reproduce these findings cannot be shared at this time because the data is also a part of an ongoing study.
